# Hakin-1, a New Specific Small-Molecule Inhibitor for the E3 Ubiquitin-Ligase Hakai, Inhibits Carcinoma Growth and Progression

**DOI:** 10.3390/cancers12051340

**Published:** 2020-05-23

**Authors:** Olaia Martinez-Iglesias, Alba Casas-Pais, Raquel Castosa, Andrea Díaz-Díaz, Daniel Roca-Lema, Ángel Concha, Álvaro Cortés, Federico Gago, Angélica Figueroa

**Affiliations:** 1 Epithelial Plasticity and Metastasis Group, Instituto de Investigación Biomédica de A Coruña (INIBIC), Complexo Hospitalario Universitario de A Coruña (CHUAC), Sergas, Universidade da Coruña (UDC), 15006 A Coruña, Spain; oami@hotmail.es (O.M.-I.); alba.casas.pais@sergas.es (A.C.-P.); raquel.carballo.castosa@sergas.es (R.C.); andrea.diaz.diaz@sergas.es (A.D.-D.); daniel.roca.lema@sergas.es (D.R.-L.); 2 Pathology Department and A Coruña Biobank from Instituto de Investigación Biomédica de A Coruña (INIBIC), Complexo Hospitalario Universitario de A Coruña (CHUAC), Sergas, Universidade da Coruña (UDC), 15006 A Coruña, Spain; angel.concha.lopez@sergas.es; 3 Computational Chemistry-UK, RD Platform Technology & Science, GSK Medicines Research Centre, Hertfordshire SG12 0DP, UK; alvarocortes@gmail.com; 4 Area of Pharmacology, Department of Biomedical Sciences and “Unidad Asociada IQM-CSIC”, School of Medicine and Health Sciences, University of Alcalá de Henares, 28805 Alcalá de Henares, Spain; federico.gago@uah.es

**Keywords:** anticancer therapy, small-molecule inhibitor, epithelial-to-mesenchymal transition (EMT), E-cadherin, E3 ubiquitin-ligase, Hakai

## Abstract

The requirement of the E3 ubiquitin-ligase Hakai for the ubiquitination and subsequent degradation of E-cadherin has been associated with enhanced epithelial-to-mesenchymal transition (EMT), tumour progression and carcinoma metastasis. To date, most of the reported EMT-related inhibitors were not developed for anti-EMT purposes, but indirectly affect EMT. On the other hand, E3 ubiquitin-ligase enzymes have recently emerged as promising therapeutic targets, as their specific inhibition would prevent wider side effects. Given this background, a virtual screening was performed to identify novel specific inhibitors of Hakai, targeted against its phosphotyrosine-binding pocket, where phosphorylated-E-cadherin specifically binds. We selected a candidate inhibitor, Hakin-1, which showed an important effect on Hakai-induced ubiquitination. Hakin-1 also inhibited carcinoma growth and tumour progression both in vitro, in colorectal cancer cell lines, and in vivo, in a tumour xenograft mouse model, without apparent systemic toxicity in mice. Our results show for the first time that a small molecule putatively targeting the E3 ubiquitin-ligase Hakai inhibits Hakai-dependent ubiquitination of E-cadherin, having an impact on the EMT process. This represents an important step forward in a future development of an effective therapeutic drug to prevent or inhibit carcinoma tumour progression.

## 1. Introduction

Carcinoma, the most common type of cancer, arises from epithelial cells. During carcinoma progression, epithelial cells acquire a high degree of plasticity and the ability to reversibly change phenotype [1,2]. Epithelial cells can undergo a program named epithelial-to-mesenchymal transition (EMT), priming their migration from the primary tumour, their dissemination and metastasis formation [3,4]. During EMT, epithelial cells acquire mesenchymal features due to the loss of cell-to-cell adhesion and apico-basal polarity, and the reorganization of actin cytoskeleton, which in turn causes the development of migratory and invasive capabilities. EMT has been associated with tumour initiation, migration, malignant progression, stemness, intravasation, metastasis, and drug resistance, with important clinical implications [5,6]. EMT is characterized by the loss of E-cadherin epithelial marker and the acquisition of mesenchymal markers, such as N-cadherin and vimentin. The loss of E-cadherin, the best characterized member of cadherins at cell–cell contacts in epithelial cells, is not only a hallmark of EMT but is also associated with the transition from adenoma to carcinoma [7,8]. The mechanisms involved in E-cadherin down-regulation and its pathological relevance have been extensively studied in cancer [9,10,11]. 

Hakai is the first posttranslational regulator of E-cadherin stability that has been described. Hakai is an E3 ubiquitin-ligase that interacts with tyrosine-phosphorylated E-cadherin, inducing its ubiquitination and degradation. This leads to disruption of cell–cell contacts in epithelial cells [12,13]. Ubiquitination is one of the most studied processes for post-translational regulation that signals for protein degradation via proteasome, lysosome or marks organelles for autophagy clearance [14,15,16]. There are three enzymatic reactions involved in the assembly of ubiquitin on substrate proteins. The E1 ubiquitin-activating enzyme is responsible for ubiquitin activation in an ATP-dependent manner. Then, ubiquitin is transferred to the E2 ubiquitin-conjugating enzyme and, finally, the ubiquitin moiety is transferred to the E3 ubiquitin-ligase, which attaches ubiquitin to the target protein to be degraded. Although ubiquitination is a multistep process, the E3 ubiquitin-ligase enzyme is responsible for the direct binding of ubiquitin to the target protein, thus conferring substrate specificity and selectivity [17,18]. Although targeting E1, E2 and the proteasome is possible, the development of inhibitors against specific E3 ubiquitin-ligases has attracted significant attention in recent years, since they can be designed to target specific substrates without affecting others, thus avoiding broader side effects [19,20]. A large body of evidence demonstrates deregulation of E3 ubiquitin-ligases in cancer [21,22,23], including their overexpression, which correlates with poor clinical prognosis and chemoresistance. Consequently, many different strategies to target ubiquitin-ligases for the treatment of cancer and other diseases have been investigated [24,25]. To date, the FDA has approved very few drugs that target members of the ubiquitin pathway [26]. The first 20S proteasome inhibitor, bortezomib, was approved in 2003, followed about a decade later by carfilzomib (2012) and ixazomib (2015) [27,28,29,30]. However, positive results with these drugs have only been observed in haematological malignancies; in contrast, the results from clinical trials on patients bearing solid tumours have been disappointing [31,32]. 

The E3 ubiquitin-ligase Hakai is essential for E-cadherin degradation and EMT induction. Moreover, Hakai-mediated down-regulation of E-cadherin is implicated in oncogenic and/or tumour-suppressive pathways such as RACK1 and Slit-Robo [13,33,34]. Hakai was not only described to regulate EMT through its action on E-cadherin [34] and cell proliferation in an E-cadherin independent manner, but also to be implicated in cell invasion and metastasis [34,35,36,37,38,39,40,41]. Although other Hakai substrates have been proposed, the physiological significance of these interactions is not well defined yet. For instance, Hakai interacts with cortactin, and mediates its ubiquitination and degradation after phosphorylation by non-receptor tyrosine kinase Src [35,42,43]. Furthermore, a recent study reports that Hakai regulates Ajuba stability, inducing Ajuba neddylation and degradation in hepatocarcinoma [44]. Hakai is a new class of RING-finger type E3 ubiquitin-ligase, which contains a novel domain named HYB (Hakai pTyr-binding, where pTyr stands for phosphotyrosine), that is structurally different from other pTyr-binding domains of E3 ubiquitin-ligases and represents a potentially attractive new drug target for anticancer therapy [32,45,46]. By using a three-dimensional structural model of Hakai’s HYB domain, a virtual screening campaign identified several small molecules with putative affinity for this site. We demonstrate that our small-molecule compound Hakin-1 (Hakai inhibitor-1) reduces Hakai-dependent total ubiquitination and Hakai-dependent ubiquitination of E-cadherin. Hakin-1 inhibits cell proliferation, oncogenic potential and invasion, and these effects are accompanied by induction of epithelial markers and reduction of mesenchymal markers in vitro. Moreover, we support the potential therapeutic value of Hakin-1 by demonstrating its antiproliferative effect in a tumour xenograft model. In conclusion, we describe Hakin-1 as the first Hakai-targeted small-molecule inhibitor that is endowed with significant antitumour activity.

## 2. Results

### 2.1. Identification of Putative Selective Hakai Inhibitors

**Figure 1:**
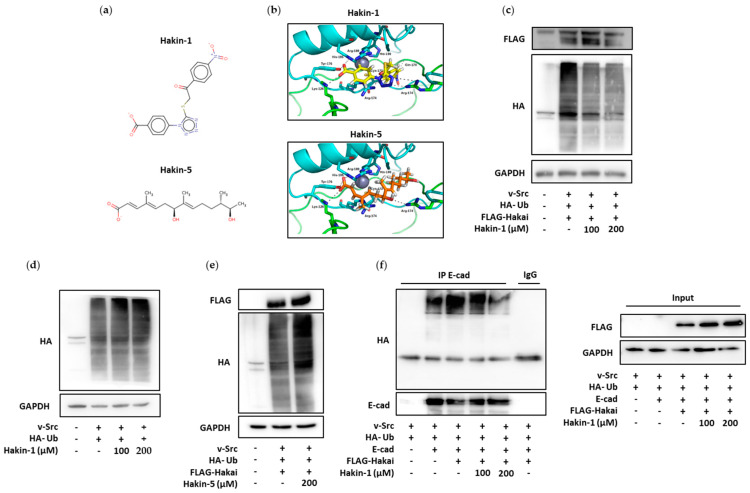
In silico and in vivo screening of E3 ubiquitin-ligase Hakai inhibitors (**a**) Chemical structure of Hakin-1 and Hakin-5. (**b**) Predicted binding poses for Hakin-1 (upper panel, in yellow) and Hakin-5 (bottom panel, in orange) molecules docked within Hakai dimers (represented in blue and green), as determined by the CRDOCK docking program. (**c**) Hakai-dependent ubiquitination assay in HEK293 cells transfected with Flag-Hakai, v-Src and HA-ubiquitin in the presence of either DMSO or compound Hakin-1. (**d**) Ubiquitination assay in HEK293T cells transfected with v-Src and HA-ubiquitin in presence of DMSO or compound Hakin-1. (**e**) Hakai-dependent ubiquitination assay in HEK293T cells transfected with Flag-Hakai, v-Src and HA-ubiquitin in the presence of either DMSO or compound Hakin-5. (**f**) Effects of Hakin-1 on Hakai-dependent ubiquitination of the E-cadherin complex. Flag-Hakai, myc-E-cadherin, v-Src and HA–ubiquitin were transiently transfected into HEK293 cells. Immunoprecipitation was performed with the anti-E-cadherin antibody before Western blotting using the indicated antibodies.

With the aim of finding candidate molecules with the required potential to inhibit Hakai, we designed a virtual screening workflow based on the structural information available and the nature of the pTyr-binding pocket, which was explored with the aid of affinity probes [35]. As a first step, we considered only molecules in our chemical library that display a negatively charged carboxylate or phosphate group that would be complementary to the highly positive molecular electrostatic potential of the binding pocket. The selected molecules were docked into the Hakai dimer to evaluate all possible binding poses and then, they were ranked using the HYDE postprocessing scoring function to estimate the interaction energy of the hypothetical Hakai-inhibitor complexes. The first 20 top-ranking molecules were visually inspected and two of them were selected for subsequent experimental validation, namely Hakin-1 and Hakin-5 (Figure 1a). According to our binding mode model, the benzoate moiety present in Hakin-1 would be a surrogate of pTyr (Figure 1b, upper panel). The carboxylate group is placed in an extremely favourable region for a negatively charged probe, as befits matching to the area highlighted by our previously calculated affinity maps. This region is lined by residues Lys-126, Tyr-176, His-185 and Arg-189. The phenyl ring, in turn, would get sandwiched between the guanidinium side chains of Arg-174 and Arg-189 and be stabilized by cation–π interactions. The rest of the molecule would further adapt to the binding pocket by establishing hydrogen bonds with the side chains of Arg-174 from both monomers and the Gln-170 backbone. Hakin-5 (Figure 1b, lower panel), despite bearing a carboxylate group and presenting an equivalent number of potential groups for hydrogen bonding interactions, lacks a phenyl ring that could mimic a pTyr and is predicted to have lower affinity (Figure S1).

### 2.2. Effect of Hakin-1 on Hakai-Induced Ubiquitination

We first investigated the effect of Hakin-1 on the enzymatic activity of E3 ubiquitin-ligase Hakai in cultured tumour cells. HEK293T cells were transfected with Src (in order to induce tyrosine phosphorylation of the E-cadherin complex) together with Hakai and ubiquitin in the presence of Hakin-1 or DMSO as control (vehicle). Hakin-1 strongly reduced the ubiquitination mediated by Hakai in a dose-dependent manner (Figure 1c) while no effect was observed in Hakai protein levels. However, Hakin-1 did not affect ubiquitination when Hakai was not overexpressed, confirming that Hakin-1 reduced the ubiquitination in a Hakai-dependent manner (Figure 1d). Moreover, no effect was detected on Hakai-mediated ubiquitination when cells were treated in the presence of Hakin-5, (Figure 1e), further supporting the specific effect of Hakin-1 on Hakai-induced ubiquitination. Finally, we observed a reduction of Hakai-dependent ubiquitination of the E-cadherin complex when cells were treated with Hakin-1 (Figure 1f). Collectively, our data show that Hakin-1 inhibits Hakai-mediated ubiquitination without affecting Hakai protein levels. 

### 2.3. Hakin-1 Activates Epithelial Cell Differentiation in Tumour Cells

**Figure 2:**
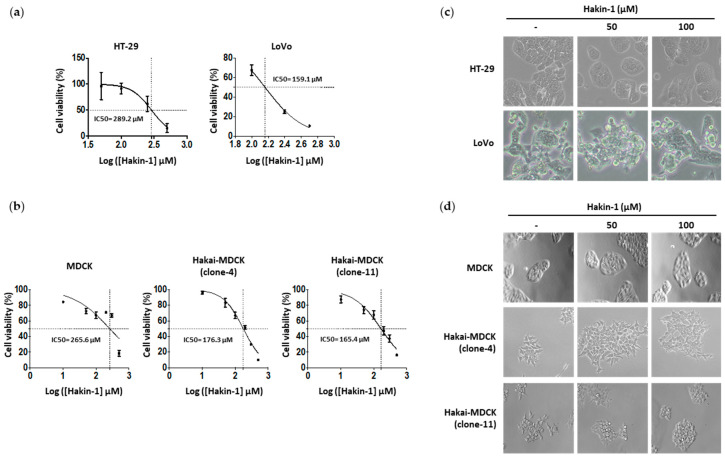
Hakin-1 induces cytotoxicity and an epithelial phenotype on epithelial tumour cell lines. (**a**) HT-29 and LoVo cells were treated with an increasing range of concentrations of Hakin-1 and cell viability was measured by means of the MTT assay (3-(4,5-dimethylthiazol-2-yl)-2,5-diphenyltetrazolium bromide). This assay was performed in six replicates and represented as mean ± SD of three independent experiments. (**b**) Cell viability was measured as indicated in panel (**a**), for MDCK and Hakai-MDCK cell lines (clone 4 and clone 11) using Hakin-1. (**c**,**d**) Phase-contrast images of HT-29 and LoVo cell lines (**c**) and MDCK, Hakai-MDCK cell lines, clone 4 and clone 11 (**d**) under Hakin-1 treatment. Images were obtained using a 20× objective.

**Figure 3:**
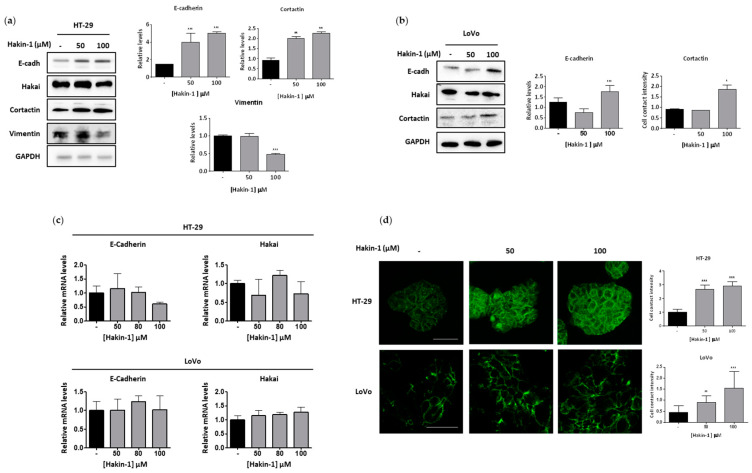
Hakin-1 induces mesenchymal-to-epithelial transition in epithelial tumour cell lines. (**a**) Western blotting analyses of epithelial-to-mesenchymal transition (EMT) markers upon Hakin-1 treatment in HT-29 cell line (left panel), and quantification by densitometry are shown (right panel). (**b**) Western blotting analyses of EMT markers upon Hakin-1 treatment in LoVo cells (left panel), and quantification by densitometry are shown (right panel). GAPDH was used as the loading control. Data show the average of three independent experiments and are represented as mean ± SD (* *p* < 0.05; ** *p* < 0.01; *** *p* < 0.001). (**c**) Hakai and E-cadherin mRNA expression levels normalized to control. RPL13A mRNA were measured in HT-29 and LoVo cells treated with Hakin-1 for 48 h. (**d**) Immunofluorescence of E-cadherin in HT-29 and LoVo cell lines in the presence of DMSO or Hakin-1 treatment after 48 h. Images were obtained with a 20× objective for HT-29 cells and a 40× objective for LoVo cells. Quantification was performed with ImageJ programme and results are expressed as mean ± SD of three independent different experiments (** *p* < 0.01; *** *p* < 0.001). Scale bar, 50 µm for HT-29 cells and 175 µm for LoVo cells.

Next, we studied the effect of Hakin-1 on cancer cell viability. We observed a dose-dependent effect of Hakin-1 on the viability of HT-29 and LoVo colon cancer cell lines (Figure 2a). In contrast, no inhibitory effect was detected when treating the same cells with Hakin-5 (Figure S2). Furthermore, we previously showed that Hakai overexpression in immortalized epithelial MDCK cells (Hakai-MDCK) induced a fibroblastic-like phenotype accompanied by the loss of E-cadherin-based cell–cell contacts [38,40]. Hakin-1 treatment was more effective at reducing the proliferation of Hakai-MDCK clones than in control MDCK nontransformed epithelial cells without overexpression (Figure 2b). These results further suggest that Hakin-1 may be particularly more cytotoxic against cancer cells that overexpress Hakai, accompanied by a slightly increased apoptosis in vitro (Figure S3). Based on the role of Hakai inducing EMT, we analysed the effect of Hakin-1 on the epithelial phenotype. By phase contrast microscopy, we observed that Hakin-1 modestly increased the epithelial phenotype of HT-29 and LoVo cells (Figure 2c). Moreover, we observed an induction of an epithelial phenotype, increased cell–cell contacts, and reduction of cytoplasmic protrusions formation, in Hakai-MDCK but not in control cells (Figure 2d). On the contrary, no effect was observed upon Hakin-5 treatment (Figure S4). We further studied the effect of Hakin-1 on the reversion of EMT markers. Increased E-cadherin epithelial marker and reduced vimentin levels were detected in Hakin-1 treated HT-29 cells (Figure 3a). We also detected that Hakin-1 increased cortactin protein, another described substrate of Hakai ubiquitination [42]. These results were confirmed in LoVo colon cancer cells (Figure 3b). Moreover, in both HT-29 and LoVo cell lines, a slight but not statistically significant decrease in Hakai expression after Hakin-1 treatment was detected (Figure S5).

Moreover, Hakin-1 did not modulate the mRNA levels of E-cadherin or Hakai confirming that its activity is mostly to control target protein degradation (Figure 3c). Hakin-1 increased the amount of E-cadherin levels at cell–cell contacts in HT-29 and LoVo cells, as detected by immunofluorescence (Figure 3d). However, no effect was detected on protein levels or localization of E-cadherin upon Hakin-5 treatment in HT-29 cells (Figure S6). Finally, we observed that Hakin-1 did not increase E-cadherin expression in Hakai-MDCK cells which, as previously reported, had a complete lack of E-cadherin basal levels [38,41]. Taken together, these results demonstrate that Hakin-1 induces epithelial differentiation in different tumour cells that is accompanied by a reduction of mesenchymal markers.

### 2.4. Hakin-1 Inhibits Proliferation, Oncogenic Potential and Invasiveness of Epithelial Tumour Cells 

**Figure 4:**
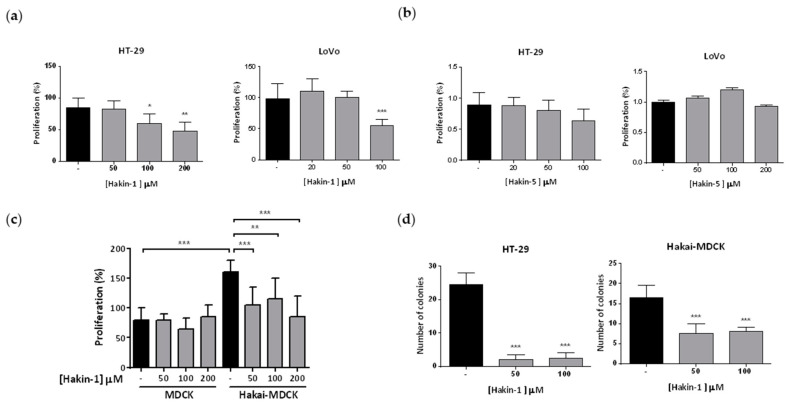
Antiproliferative and antioncogenic effect of Hakin-1 in tumour epithelial cells. (**a**) HT-29 and LoVo cells were treated with Hakin-1 for 48 h and proliferation was measured by performing a BrdU assay as indicated in Material and Methods. Results are expressed as mean ± SD of eight replicates and experiments were repeated three times (* *p* < 0.05; ** *p* < 0.01; *** *p* < 0.001). (**b**) HT-29 and LoVo cells were treated with Hakin-5 for 48 h and proliferation was measured as indicated in (**a**). (**c**) MDCK and Hakai-MDCK cells were treated with increasing concentrations of Hakin-1 for 48h and proliferation was measured as indicated in (**a**). (**d**) Soft agar assay in HT-29 (left panel) and Hakai-MDCK (right panel) cell lines. Colonies grew for 28 days (HT-29) or 21 days (Hakai-MDCK) and were counted as indicated in Materials and Methods. Quantification of the colonies was performed in triplicates and represented as mean ± SD of three independent experiments; (*** *p* < 0.001).

**Figure 5:**
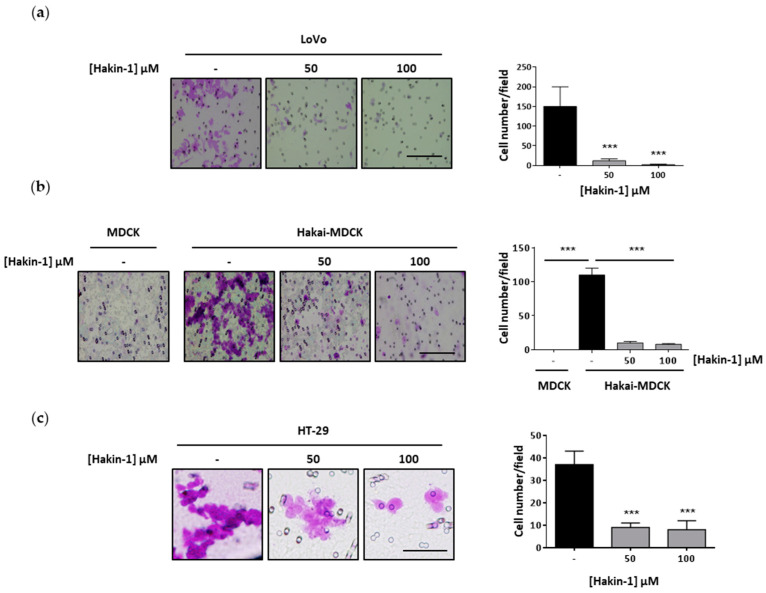
Hakin-1 reduces cell invasion and cell migration of epithelial tumour cells. (**a**) Invasion assay in LoVo cell line was performed as described in Materials and Methods. Cells were treated in the presence of DMSO or Hakin-1 for 48 h before being seeded into an invasion chamber. Representative images were taken using the 20× objective (right panel) and quantification of the photographed invasive cells are shown (left panel). (**b**) Invasion assay was performed as previously indicated by using MDCK and Hakai-MDCK cells. (**c**) Migration assay in HT-29 cells was analysed after treatment with DMSO or Hakin-1 during 48 h. Cells were seeded in a migration chamber as described in Materials and Methods. Representative images taken with 20× objective (right panel) and quantification of migrating cells (left panel) are shown. Results are represented as mean ± SD of triplicates of three independent experiments (*** *p* < 0.001).

Given that Hakai affects not only cell–cell contacts but also proliferation in fibroblast and epithelial cells [38], we decided to determine the possible effect of Hakin-1 on proliferation. Indeed, Hakin-1 (Figure 4a) but not Hakin-5 (Figure 4b) reduced cell proliferation in HT-29 and LoVo cells. Moreover, we confirmed that MDCK cells strongly proliferated when Hakai was overexpressed (Figure 4c). Interestingly, Hakin-1 was able to suppress proliferation of Hakai-MDCK cells whereas MDCK control cells were unaffected (Figure 4c). These results suggest that Hakin-1 may function as an antiproliferative agent when Hakai is highly expressed in epithelial cells, as observed in tumours from colorectal cancer patients [39,45,47]. Hakin-1 also inhibits cell proliferation in other epithelial cells lines such as breast cancer MCF7 cells, prostate cancer PC3 cells, bladder cancer 5637 cells, renal cancer ACHN cells and liver cancer HepG2 cells (Figure S7). We also observed a significant reduction of colony formation in soft agar upon treating HT-29 and Hakai-MDCK cells with Hakin-1 (Figure 4d). As we previously described, MDCK nontransformed cells do not form colonies, thus no effect was detected upon Hakin-1 treatment [38]. As stated above, the EMT process is characterized by the acquisition of migratory and invasive capabilities. We demonstrated that Hakin-1 strongly reduced the invasion capacity of LoVo cancer cells (Figure 5a). Moreover, we show that Hakin-1 blocked the invasion induced by Hakai overexpression in MDCK cells (Figure 5b). Finally, given that HT-29 cells were unable to invade under these experimental conditions, the effect of Hakin-1 on cell motility was tested and an important reduction of cell migration was observed (Figure 5c). All of these findings support an in vitro antitumour effect of Hakin-1 by acting on cell proliferation, oncogenic potential, cell motility and invasiveness.

### 2.5. Antitumour Effect of Hakin-1 in Tumour Xenografts In Vivo

**Figure 6:**
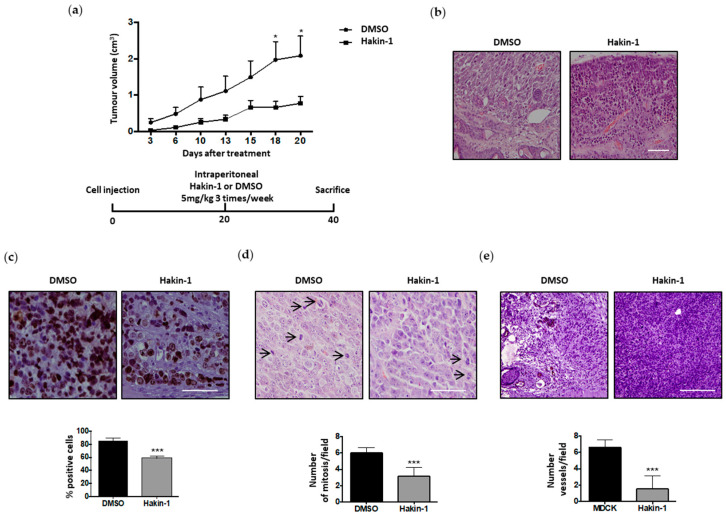
Hakin-1 inhibits tumour growth in xenografted mice. (**a**) Hakin-1 effect on tumour growth in nude mice inoculated into the flank with Hakai-MDCK cells (n = 8 tumours). Tumour growth curve is shown in the upper panel. Error bars represented the mean ± SEM (* *p* < 0.05). A schematic representation of the experiment design is shown in the bottom panel. (**b**) Haematoxylin and eosin (H&E) staining of Hakai-MDCK tumours at the end point, treated with DMSO (left panel) or Hakin-1 (right panel). Images were obtained with a 20× objective. Scale bar, 300 µm. (**c**) Immunohistochemistry of Ki67 marker in Hakai-MDCK tumours treated with DMSO (left panel) or Hakin-1 (right panel). Representative images were obtained with 40× objective. Scale bar, 500 µm. Quantification of the percentage of positive cells is shown in the bottom panel. (**d**) Representative image of Hakai-MDCK tumours in nude mice stained with H&E is shown. Pictures were obtained with a 40× objective (upper panel). Quantification of the number of mitosis (arrows) in high magnification field is shown (bottom panel). Scale bar, 500 µm. (**e**) Immunohistochemistry of CD31 marker in Hakai-MDCK tumours of injected nude mice treated with DMSO (left panel) or Hakin-1 (right panel). Images were obtained with a 20× objective. Scale bar, 500 µm. Quantification of the number of fields is shown in the bottom panel and is expressed as mean ± SEM (*** *p* < 0.001).

The acquisition of migratory and invasive capabilities during EMT are crucial events in the formation of distant metastases, therefore targeting these events is an ideal approach for cancer treatment [1,2,3,4]. Based on our results with Hakin-1 blocking cell proliferation, oncogenic potential, invasiveness and motility of cancer cells in culture, we decided to study its efficacy on tumour growth in vivo. Control-MDCK and Hakai-MDCK cell were subcutaneously injected into the flank of nude mice. As previously reported, Hakai-MDCK cells formed primary tumours whereas parental MDCK cells did not [41]. Hakin-1 displayed a potent effect on inhibiting xenograft tumour growth (Figure 6a). Morphologically, xenograft tumour cells exhibited an undifferentiated and spindle-shape phenotype, a large nucleus and a small cytoplasm size. This morphology was strongly altered by Hakin-1 treatment, inducing tumour cell differentiation and an increase in cytoplasmic size (Figure 6b). Furthermore, by analysing Ki67 and the mitotic index, we show that Hakin-1 markedly reduced the number of proliferative cells (Figure 6c,d), whereas no effect was detected on apoptosis (Figure S8). These results underscore Hakin-1’s effect on cell proliferation inhibition in vivo. We also detected a strong reduction in the number of blood vessels in tumour xenografts upon Hakin-1 treatment by staining for the CD31 endothelial marker (Figure 6e). Interestingly, no damage was observed in liver and kidney tissues in Hakin-1-treated nude mice (Figure S9), which showed a normal morphological structure, supporting that Hakin-1 treatment inhibits tumour growth in nude mice apparently without systemic toxicity. 

### 2.6. Hakin-1 Treatment Reduces N-Cadherin Mesenchymal Marker in Tumour Xenografts and Initiation of Lung Metastases

**Figure 7:**
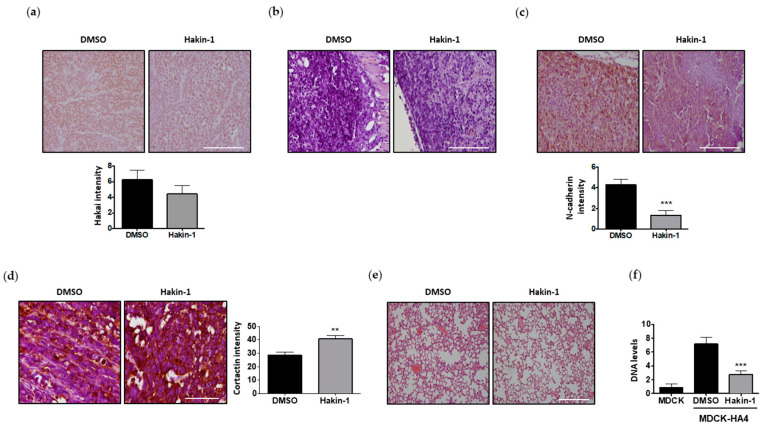
Hakin-1 treatment reduces mesenchymal markers of tumour xenografts and micrometastasis formation in lungs of immunosuppressed mice. (**a**–**c**) Immunohistochemical staining for Hakai (**a**), E-cadherin (**b**) and N-cadherin (**c**). Representative images were obtained with a 20× objective (upper panel). Quantification of significantly protein expression intensity is shown in bottom panel. (**d**) Immunohistochemical staining for cortactin and protein expression quantification is shown (left and right panels, respectively). Images were obtained with 40× objective. (**e**) H&E staining of mice lungs did not show a histological evidence of metastases. Representative images were obtained with a 10× objective. (**f**) Real-time quantitative PCR using primers for HA epitope and Hakai to detect the presence of DNA of Hakai-MDCK cells into the mice lungs. Results are expressed as mean ± SEM (*** *p* < 0.001). Scale bar, 500 µm.

We further evaluated the in vivo effect of Hakin-1 on the reversion of EMT, as a crucial process in tumour progression and invasion. First, we confirmed that the levels of Hakai protein were not affected by Hakin-1 in tumour xenografts growing in nude mice (Figure 7a). As no E-cadherin protein was detected in Hakai-MDCK cells, we did not detect E-cadherin in tumour xenografts either in the presence or the absence of Hakin-1 (Figure 7b). However, we observed a strong reduction of N-cadherin, a mesenchymal marker of EMT, upon Hakin-1 treatment in tumour xenografts (Figure 7c). These data further support that Hakin-1 reverts the cell mesenchymal phenotype by a reduction of N-cadherin EMT mesenchymal marker. In consonance with this, we also analysed the effect of Hakin-1 on cortactin, another well-described substrate for Hakai. Hakai mediates the interaction and ubiquitination of cortactin protein inducing its degradation [42]. As shown in Figure 7d, cortactin is increased in Hakai-MDCK tumour xenografts upon Hakin-1 treatment. This result further supports the capacity of Hakin-1 to inhibit the ubiquitination and degradation of cortactin by Hakai. To determine whether Hakin-1 may affect cancer metastasis, lung tissues from nude mice were analysed by haematoxylin and eosin (H&E) staining. Nevertheless, after an exhaustive analysis, distant metastases were not detected under the experimental conditions (Figure 7e). Finally, we detected that Hakin-1 caused a significant reduction of micrometastasis formation in lungs of mice subcutaneously injected with Hakai-MDCK cells (Figure 7f). Metastases were not detected by histological H&E staining but by amplifying exogenous Hakai using PCR on lung tissue DNA, using HA-epitope as the first primer and Hakai as the second primer. These results demonstrate the capacity of Hakin-1 to inhibit lung metastasis in vivo.

## 3. Discussion

Epithelial plasticity is a well-documented and a crucial event for efficient invasion and metastasis, with relevant clinical implications [5,6]. As an important precondition of metastasis, the EMT process has become a promising target for anticancer therapy. In our search for small-molecule inhibitors directly targeted against EMT, we focused our attention on the E3 ubiquitin-ligase Hakai, a post-translational regulator that induces E-cadherin degradation, whose loss at cell–cell contacts is a major hallmark of EMT. In this study, a virtual screening campaign identified several small molecules inhibitors with potential affinity for HYB domain of Hakai, a novel pTyr-binding domain for E3 ubiquitin-ligases that targets specific substrates, such as phosphorylated E-cadherin [35,45]. We address several important points. First, we demonstrate that Hakin-1 is the first reported inhibitor that reduces Hakai-mediated total ubiquitination and Hakai-mediated ubiquitination of E-cadherin in a dose-dependent manner. Second, Hakin-1 significantly inhibits epithelial cancer cell viability and proliferation, showing much lower cytotoxic and antiproliferative effect in normal epithelial cells with lower levels of Hakai; this suggests that it may be particularly more effective when Hakai is highly expressed. Third, Hakin-1 increases epithelial differentiation, which is accompanied by an increased expression of Hakai substrate, E-cadherin at cell–cell contacts, and a reduction of vimentin mesenchymal markers. These findings support that Hakin-1 reverts the EMT process in vitro. Fourth, Hakin-1 also reduces important features of EMT in vitro, such as migration and invasiveness. Fifth, Hakin-1 decreases tumour growth in a xenograft mouse model without apparent systemic toxicity. Finally, Hakin-1 reduces N-cadherin mesenchymal markers in tumour xenografts and lung micrometastasis in vivo. Therefore, we postulate that Hakin-1 is an effective antiproliferative agent that can inhibit EMT, at least in part, through its specific action on Hakai-mediated ubiquitination and degradation of E-cadherin. To date, there is an extended interest in developing EMT inhibitors targeting different signalling pathways or stimuli related to EMT in order to block or prevent tumour progression and metastasis [47]. It is proposed that anti-EMT drugs are expected to preferentially block metastasis initiation and drug resistance rather than tumour growth. Unfortunately, the clinical activity of most EMT inhibitors has been exclusively evaluated based on their capacity to reduce tumour growth. This is mostly due to the fact that EMT-related inhibitors were not initially developed to directly block EMT [47]. In consequence, conventional clinical trial designs have underestimated the antitumour potential of anti-EMT drugs that may not significantly impact on tumour growth but rather in metastasis formation and drug resistance. Interestingly, although it is unknown how Hakin-1 may reduce the number of blood vessels in tumour xenografts, a recent publication demonstrated that soluble E-cadherin promotes tumour angiogenesis and localizes to exosome surface in ovarian cancer [48]. Given that Hakin-1 inhibits the degradation of E-cadherin and increases its localization at cell–cell contacts, we could speculate that Hakin-1 could reduce the amount of soluble E-cadherin that is internalized into exosomes. This hypothesis opens up a new line of investigation to explore the possible antiangiogenic effects of Hakin-1. In addition, given that Hakai was described not only as a key regulator of EMT, by its action on E-cadherin degradation, but also as a regulator of cell proliferation, Hakin-1 becomes the first small-molecule compound that directly affects EMT by its action on Hakai, thus being a great candidate for future clinical development.

However, although Hakin-1 regulates Hakai-mediated ubiquitination and degradation of E-cadherin, it may also exert its action on additional specific Hakai substrates that have not been discovered yet. This unexplored possibility merits further investigations. Furthermore, although Hakin-1 effect are, at least in part, mediated by its action on Hakai, possible off-target effects cannot be ruled out as Hakin-1 could also bind to and inhibit other yet unidentified E3 ubiquitin-ligases expressed in epithelial cells that share the HYB domain, such as the testis-specific E3 ubiquitin-ligase ZNF645, also known as CBLL2 [35]. In fact, our bioinformatic analysis reveals a high degree of sequence identity and structural conservation between these two targets (Figure S1). Future investigations will aim to more deeply evaluate its effect as an anti-EMT drug that inhibits metastasis. Further work is also required to determine how Hakin-1 may impact on angiogenesis, mediated by other potentially druggable targets. On the other hand, it was reported that early stages of EMT involve a post-translational downregulation of E-cadherin, whereas loss of E-cadherin via transcriptional repression is a late event in EMT [49,50]. Recently, it has been proposed that novel compounds with the ability to potentiate the activity of FBXL14, a convergent E3 ubiquitin-ligase that controls protein stability of the EMT transcription factors Snail, Slug, and Twist, could also make up another class of plausible anti-EMT therapeutic agents [51]. In this regard, inhibition of the Dub3 deubiquitinase by its specific inhibitor WP1130 has been reported to suppress breast cancer invasion and metastasis by promoting Snail1 degradation. All these data open a new field of investigation of searching for inhibitors that act post-translationally to control EMT [52]. 

On the other hand, although Hakai was the first reported post-translational regulator of E-cadherin stability, MDM2, a RING-finger type E3 ubiquitin-ligase, also induces E-cadherin ubiquitination and degradation [53]. Considerable efforts have been made to target MDM2, given that it also mediates the degradation of p53 tumour suppressor. Indeed, an important number of compounds, including Nutlin-3, have been identified to suppress the p53-MDM2 interaction [54,55]. Nutlin-3 and other analogues were tested in preclinical assays and/or registered for phase I trial for an array of malignancies [56]. In this regard, Nutlin-3 abolishes E-cadherin downregulation through TGF-β in p53-deficient tumour cells, by blocking Smad2 and Smad3 phosphorylation, and subsequently decreased Snail and Slug transcription [57]. In contrast, our results show a direct effect of Hakin-1 on E-cadherin ubiquitination and degradation mediated by Hakai. Later on, a novel compound was reported to directly bind to MDM2 (SP-141) and inhibits its protein levels by inducing autoubiquitination and proteasome degradation, independently of the p53 status. SP-141 also affects EMT-related proteins, such as β-catenin, vimentin, Twist and Snail and suppresses breast cancer migration in vitro and metastasis in vivo. However, no direct effect on E-cadherin expression was observed in MDA-MB-231 breast cancer cells. Therefore, to the best of our knowledge, Hakin-1 is the first reported inhibitor of an E-cadherin post-translational regulator that has been specifically selected for its affinity for the binding pocket wherein phosphorylated-E-cadherin specifically binds and influences EMT.

Important publications propose MDM2 as a promising therapeutic drug to treat human cancer, including breast cancer. Indeed, MDM2 is a negative prognostic marker and it has been shown that high MDM2 protein levels are detected in tumour biopsies from breast cancer patients with lymph node metastases [58]. Similarly, Hakai has been proposed as a novel biomarker for colon cancer progression [39,41]. Hakai protein expression gradually increases in human colon adenocarcinoma (TNM-stages I–IV) compared to adjacent healthy colon tissue, and statistically significant differences are indeed detected in colon adenoma compared to healthy tissue. Given that we show that Hakin-1 exerts an antitumour activity in LoVo and HT-29 colon cancer cells, by inhibiting proliferation, colony formation, migration and invasiveness, accompanied by an increase of E-cadherin expression at cell–cell contacts, we propose that Hakin-1 has promising therapeutic potential against colon cancer growth and progression. 

The development of inhibitors against specific E3 ubiquitin-ligases has attracted significant attention, since they can be designed to target specific substrates without affecting others, thus avoiding broader side effects. Remarkably, the observed antitumour effect of Hakin-1 in tumour xenografts was noticeable even at a low dose of 5 mg/kg, only used every three days. Moreover, by histological examination of several tissues and the body weight control in the mouse, we did not detect any toxicity upon Hakin-1 administration. Therefore, it seems plausible to further optimize the doses, frequency or formulation of Hakin-1 in order to abolish tumour growth and metastasis completely.

As we previously mentioned, to date, the FDA has so far approved three drugs designed to target the ubiquitin pathway, including the proteasome inhibitors bortezomib, carfilzomib and ixazomib. Moreover, the proved efficacy of these drugs is for haematological malignancies, while clinical activity in solid tumours has been limited [27,28,59]. Since epithelial cells undergoing EMT reported to show decreased proteasome activity, a potential risk of proteasome inhibitor-based therapy in this type of malignancies has been noted [42,60]. Interestingly, we show that Hakin-1 inhibits cell proliferation in different epithelial cell lines, while no inhibition was detected in haematological tumour cells. Therefore, we propose that the use of Hakai-inhibitors targeting the ubiquitin pathway might be a better therapeutic strategy in epithelial tumours undergoing EMT.

Cancer metastasis consists of a sequential series of events including EMT and the reverse process known as mesenchymal-to-epithelial transition (MET). MET is necessary for metastatic colonization once malignant cells find their niche in distant organs. Therefore, this cellular plasticity during EMT has to be taken into consideration when analysing the contribution of EMT to cancer, as different degrees of epithelial plasticity may be present. In consequence, the translation of the EMT paradigm into the clinic represents an important challenge and remains as a controversial issue [47,61]. With the aim of identifying compounds acting on EMT, it is extremely meaningful to search for inhibitors that could block metastasis at the early stage, in order to prevent or inhibit EMT before metastases have taken place. In this regard, compounds acting on mesenchymal-type cells may accelerate tumour cell growth at distant metastasis because of MET. Interestingly, we observed that Hakin-1 treatment reduced tumour growth in Hakai-MDCK tumour xenografts, on which E-cadherin was not expressed, and this effect was accompanied by a reduced expression of N-cadherin mesenchymal marker and reduced formation of lung micrometastases. Future investigations will help to elucidate the beneficial effect of Hakin-1 in human colon cancer treatment. In conclusion, Hakin-1 emerged as an effective therapeutic agent for EMT inhibition with therapeutic potential and our results constitute the first preclinical proof-of-concept that Hakai inhibitors could be useful as anticancer agents.

## 4. Materials and Methods

### 4.1. Protein and Ligands Models

The X-ray crystal structure of the pTyr-binding domain of Hakai (PDB 3VK6) [35] was downloaded from the Protein Data Bank and the dimer modelled using the proper symmetry operations. Amino acid protonation was carried out using the pdb2pqr server [62] at a pH of 7.2. 3D models for the ligands were built using the virtual screening and data management integrated platform (VSDMIP), as described elsewhere [63,64]. Briefly, the initial 3D coordinates for each ligand were generated with CORINA. Thereafter, ALFA [65] was used to generate a large variety of conformers for each of which MOPAC-calculated atomic partial charges were assigned by employing the AM1 semiempirical model and the ESP method. All ligand models were stored in the VSDMIP database to be used in the different virtual screening campaigns.

### 4.2. Virtual Screening

Ligands in the eMolecules catalogue (https://www.emolecules.com/info/products-screening-compounds.html) were downloaded and processed as described in the preceding section. Only molecules presenting a carboxylic acid moiety and/or a phosphate group capable of mimicking a pTyr residue were considered. Next, CRDOCK [66] was used to lodge the selected molecules inside the binding pocket of Hakai by using the CRScore scoring function and the BFGS energy minimizer. The ligands were then ranked according to the predicted score and the top 350 molecules were re-evaluated by using an in-house implementation of the HYDE scoring function [60]. Finally, the best 20 molecules were visually inspected to select a final set of six prospective candidates.

### 4.3. Binding Pocket Analysis

To better analyse the results of the virtual screening campaign, we used our in-house cGRILL software [67] to produce affinity maps within the binding pocket of Hakai’s pTyr-binding domain based on the van der Waals, Coulombic and hydrogen bonding interactions of hypothetical atomic probes. The negatively charged acceptor probe (=O) was used to map possible locations for the molecular recognition of the pTyr residue to help filter the docking solutions during the visual inspection of the poses.

### 4.4. Plasmids, Inhibitors and Antibodies

pcDNA-Flag-Hakai, pBSSR-HA–ubiquitin, pSG-v-Src and pcDNA-myc-E-Cadherin plasmids were previously described. Compounds Hakin-1 [4-(5-{[2-(4-nitrophenyl)-2-oxoethyl]thio}-1H-tetrazol-1-yl)benzoic acid] and Hakin-5 [(2E,4E,8E)-7,13-Dihydroxy-4,8,12-trimethyl-2,4,8-tetradecatrienoic acid] were obtained from ChemBridge Corporation and TimTec or Analyticon Discovery, respectively. Compounds were resuspended in DMSO (Sigma Aldrich, St Louis, MO, USA) at 100 mM for in vitro and in vivo assays. The highest concentration of DMSO was used as the vehicle control for the experiments. The antibodies used are listed in Supplementary Materials.

### 4.5. Cell Culture

MDCK, HEK293T, HEK293, HepG2, MCF7, and ACHN cells were cultured in Dulbecco´s modified Eagles medium (DMEM). MDCK stably expressing Hakai cells (Hakai-MDCK) were previously reported and were growth in DMEM with G418 (800 µg/mL) [38]. Different clones of Hakai-MDCK cells shown comparable phenotypes and characteristics as demonstrated previously [38]. LoVo and PC-3 cells were cultured in Ham´s F-12 Medium and HT-29 cells in McCoy’s 5A medium. 5637 cells were cultured in RPMI medium. All culture media were supplemented with 1% penicillin/streptomycin and 10% of heat-inactivated foetal bovine serum (FBS) at 37 °C in a humidified incubator with 5% CO_2_. Cells were monthly tested for mycoplasma contamination and used only for 1–3 months after defrosted. LoVo and HT-29 cells were authenticated with the StemElite ID system (Promega, Madison, WI, USA). For phase-contrast images, culture cells were photographed with a Nikon Eclipse-TI microscope. 

### 4.6. Ubiquitination Assays

For ubiquitination assays, 7.5 × 10^5^ HEK293T cells were seeded in 60 mm dishes and after 24 h were transfected with 0.25 µg Src, 0.75 µg Flag-Hakai, and 0.5 µg HA-ubiquitin with Lipofectamin 2000 (Invitrogen, Carlsbad, CA, USA). Six hours after transfection, cells were treated with indicated concentrations of Hakin 1 or Hakin-5 for 36h. Whole cell extracts were obtained in lysis buffer (20 mM Tris/HCl pH 7.5, 150 mM NaCl and 1% Triton X-100) containing 10 μg/mL leupeptin, 10 μg/mL aprotinin and 1 mM phenylmethanesulphonyl fluoride (PMSF), supplemented with 10 mM N-ethylmaleimide. Cells were harvested and subjected to Western blotting using anti-HA antibody to detect ubiquitination.

### 4.7. Immunoprecipitation

For immunoprecipitation experiments, 4 × 10^6^ HEK293 cells were transfected with 3 µg Src, 4 µg Flag-Hakai, and 2 µg HA-ubiquitin and 3 µg E-cadherin with Lipofectamin 2000 (Invitrogen, Carlsbad, CA, USA). Six hours after transfection, cells were treated with indicated concentrations of Hakin-1 for 24 h. Then, cells were lysed for 20 min in 1 mL of 1% Triton X-100 lysis buffer (20 mM Tris-HCl pH 7.5, 150 mM NaCl and 1% Triton X-100) containing 10 μg/mL leupeptin, 10 μg/mL aprotinin and 1 mM phenylmethanesulphonyl fluoride (PMSF), supplemented with 10 mM N-ethylmaleimide and 2.5 mM sodium orthovanadate. After centrifugation at 18,000× *g* for 10 min, the supernatants were immunoprecipitated for 2 h with 2 μg of anti-E-cadherin antibody bound to 60 μL of protein G PLUS-Agarose beads (Santa Cruz Biotechnology, Dallas, TX, USA), followed by SDS-polyacrylamide gel electrophoresis (PAGE) and Western blotting with the indicated antibodies as previously reported.

### 4.8. Viability Assays

For cytotoxicity assays, 1 × 10^4^ cells were seeded per well into a 96-well plate. After 24 h, cells were treated with the indicated inhibitors for 72 h and an MTT colorimetric cell viability assay was performed following manufacturer’s instructions (Sigma Aldrich). Absorbance was measured at 570 and 630 nm using a Multiskan Plus Reader (Nanoquant Infinite M200 Tecan Trading AG, Switzerland). Dose-response curves were designed with GraphPad Prism Software and the half-maximal inhibitory concentration (IC_50_) values were calculated. Represented data are the mean ± SD of at least three independent experiments with eight replicates per condition.

### 4.9. Western Blotting and Immunofluorescence

For Western blot analysis, cells were treated with the indicated inhibitors for 48 h and the whole cell extracts were obtained as described previously. Twenty micrograms of lysates were resolved on a 10% polyacrylamide SDS-PAGE and Western blot analysis was performed as previously described. For immunofluorescence assays, cells were grown for 24 h on glass coverslips and treated with the indicated inhibitors for 48 h. Cells were fixed with 4% PFA for 15 min, permeabilized with 0.5% Triton X-100 and incubated with E-cadherin antibody for 2 h. Coverslips were incubated with fluorescein-tagged secondary antibody (Alexa-Fluor, ThermoFisher Scientific, Waltham, MA, USA) for 1 h. Finally, coverslips were mounted with ProLong Gold antifade reagent (LifeTech, Carlsbad, CA, USA) and images were taken in an epifluorescence microscope (Olympus) using a 40× objective.

### 4.10. Real-Time Quantitative PCR (qRT-PCR)

HT-29 and LoVo cells were treated with increasing concentrations of Hakin-1 (50, 80 and 100 µM) for 48 h. Total RNA extraction was performed using a TRIzol™ RNA isolation protocol and cDNA synthesis was carried out by RT-PCR, under the specifications of the reverse retrotranscriptase kit (NZYTech). mRNA levels were analysed in technical triplicates by real-time PCR (qPCR) in a Light Cycler 480. Comparative CT method (ΔΔCT method) was performed to analyse qPCR data. Primers used were as follow: for E-cadherin F-AGTGTCCCCCGGTATCTTCC and R-CAGCCGCTTTCAGATTTTCAT and for Hakai F-CGCAGACGAATTCCTATAAAGC and R-CCTTCTTCATCACCAGGTGG. RPL13A was used as a housekeeping using the following primers F-CAAGCGGATGAACACCAAC and R-TGTGGGGCAGCATACCTC.

### 4.11. Proliferation Assays

For BrdU assays, 1 × 10^4^ of indicated cells were plated per well into a 96-well plate. After 24 h, cells were treated with the indicated inhibitors for 48 h. Three independent experiments were plated with eight replicates per each condition. Cells were treated with 10 mM BrdU for 2 h. BrdU incorporation into newly synthesized DNA was measured using a cell proliferation colorimetric immunoassay kit according to the manufacturer’s instructions (Roche, Switzerland). Results are expressed as mean ± SD. Results are represented as percentage of positive cells (mean ± SD) of three independent experiments. 

### 4.12. Soft Agar-Colony Formation Assay

Soft agar-colony formation assay was performed on 12-well plates in triplicates at a density of 5 × 10^3^ MDCK and Hakai-MDCK cells/well, or 12 × 10^3^ HT-29 cells/well. Cells were seeded in medium with 0.5% low-melting agarose over a layer with 0.75% low-melting agarose (Lonza Rockland, ME, USA). Cells were treated with the indicated inhibitors and DMSO was used as vehicle. Treatment was refreshed every 3 days and, after 21 days for MDCK and Hakai-MDCK cells or 28 days for HT-29 cells, number of colonies were quantified. Quantification of five randomly selected fields of each condition was photographed with a Nikon Eclipse-TI microscope (objective 4×). Experiments were conducted with three triplicates and were repeated three times. Data are represented as mean ± SD.

### 4.13. Migration and Invasion Assay

For invasion assays, cells were treated with Hakin-1 or DMSO as vehicle for 48 h using 1% FBS during the last 24 h. Then, 3 × 10^5^ MDCK, Hakai-MDCK or LoVo cells were seeded in a cell invasion chamber (cell invasion assay kit, Chemicon International) containing medium with 2% FBS and after 16 h, invasive cells that reached the lower chamber containing 30% FBS were fixed and stained with crystal violet (Sigma Aldrich, St Louis, MO) following the manufacturer’s specifications. For migration assays, HT-29 cells were cultured with Hakin-1 or DMSO as vehicle for 48 h, using medium without serum for the last 24 h. 3 × 10^5^ HT-29 cells were seeded in the cell migration chamber (Cell migration kit, Millipore, Bedford, MA, USA) containing medium without serum. After 16 h, migrated cells in the lower chamber containing serum with 30% FBS were stained with crystal violet and counted following the manufacturer’s specifications. For both invasion and migration assays, cells were counted in five fields photographed with an Olympus microscope using a 20× objective. Experiments were performed in triplicates for each condition and the assays were repeated at least three times. Results are expressed as mean ± SD.

### 4.14. Tumour Xenograft Model

Xenograft experiments were performed in the Experimental Surgery Unit—Technological Training Center from INIBIC in compliance with the European Community Law (86/609/EEC) and the Spanish law (R.D. 53/2013). The experiment was approved by the Ethics Committee for Animal Experimentation of Xerencia de Xestion Integrada da Coruña (XXIAC, ethic code: 2017/R12). Mice were in a 12/12 h light/dark cycle with water and food available ad libitum. Six weeks old athymic nu/nu mice were randomly distributed in groups. One million of MDCK cells, resuspended in DMEM without serum and antibiotic, were subcutaneously inoculated in both flanks in two groups of 3 animals. The same number of Hakai-MDCK cells was injected in two groups of 4 animals. Twenty days after inoculation, tumours in Hakai-MDCK were palpable. Then, half of the animals were treated with Hakin-1 (5 mg/kg) and the other half with the same concentration of DMSO every 3 days. Tumour outgrowth was monitored twice a week taking measurements of tumour length (L) and width (W) with an electronic calypter. Tumour volume was calculated as pLW^2^/6. Forty days after inoculation, animals were euthanised. Tumours, lungs, kidneys and livers were collected and fixed in 4% PFA and embedded in paraffin blocks for histology and/or immunohistochemistry analyses.

### 4.15. Histology and Immunohistochemistry

Tumours and tissues were deparaffinised, rehydrated and stained with haematoxylin and eosin (H&E) as previously described [68]. Tumour sections (4 µm) were also deparaffinised and hydrated for immunohistochemistry. Antigen retrieval was carried by heating the samples (2100 Retriever; PickCell Laboratories, Amsterdam, The Netherlands) in citrate buffer or in EDTA buffer (Agilent, Santa Clara, CA, USA). Then, endogenous peroxidase activity was blocked with peroxidase blocking (Agilent). Samples were blocked and permeabilized with 0.2% BSA and 0.1% Tx-100 for 1 h and incubated with the indicated primary antibodies overnight at 4 °C in a wet chamber. Slides were incubated for 1 h at room temperature and the secondary antibody and detection was carried out using DAB (Dako Real Envision kit, Agilent) according to manufacturer instructions. The antibodies used are listed in Supplementary Materials. Finally, nuclei were counterstained with Gill´s haematoxylin and mounted with DePeX. Pictures were taken with an Olympus microscope. Quantification of images was performed with ImageJ software by analysing 5 photographs of each animal, and the represented results are shown as mean ± SEM. The number of mitosis was counted in sections stained with H&E. In this case, ten pictures of each tumour were taken with an Olympus BX50 microscope (objective 40×) and the number of mitosis was counted manually. Results are represented as mean ± SEM and a representative photograph is shown for each condition. 

### 4.16. Quantification of Lung Metastasis from In Vivo Mouse Model

Real-time PCR [69] was used to study the presence of metastasis in the lung mice. Primers for HA epitope and Hakai present in ectopic HA-tagged Hakai expressed in Hakai-MDCK cells (5′-TCTGGGACGTCGTATGGGTA-3′; 5′-TTCTTCATCACCTTGCGGG-3′) were used for the quantification. Primers for mouse apolipoprotein B (apob) (5′- CGTGGGCTCCAGCATTCTA-3′; 5′- TCACCAGTCATTTCTGCCTTTG-3′) were used as endogenous control. MDCK cell line was used as negative control. Lung DNA was extracted from 10–15 sections of paraffin blocks (4 µm) using the QIAamp DNA Mini Kit (Qiagen) as previously described [70]. The amplification and quantification of DNA was carried by quantitative PCR in technical triplicates by using a Light Cycler 480 real-time light cycler (Roche). Relative DNA levels were calculated by 2^−ΔΔCt^ method. 

### 4.17. Statistical Analysis

Statistical analyses were carried out by using GraphPad Prism software. The Shapiro–Wilk test was used to check a normal distribution and Levene test to assess the equality of variances. Statistical significance of data was determined with ANOVA with the Bonferroni test or Kruskal–Wallis with the Tukey correction test. Significance among the experimental groups indicated in the figures is shown as * *p* < 0.05, ** *p* < 0.01 and *** *p* < 0.001. Results obtained are expressed as mean ± SD or mean ± SEM as indicated. Results are represented as fold induction of treated cells over the values obtained in the untreated cells.

## 5. Conclusions

To date, the FDA has approved very few drugs targeting the ubiquitin pathway, and they were only authorised for haematological malignancies. However, the inhibitors designed against this pathway show limited clinical benefit in solid tumours. On the other hand, the epithelial-to mesenchymal transition (EMT) process is considered as a promising therapeutic target to block cancer progression before metastases have taken place. We demonstrate that Hakin-1 is an effective inhibitor against the E3-ubiquitin-ligase Hakai that targets E-cadherin for degradation, a hallmark of EMT, showing a potent antitumour effect in vitro and in vivo in epithelial tumours. Our results represent an important step forward in a future development an effective therapeutic drug to prevent or inhibit metastasis that may benefit patients with a carcinoma.

## 6. Patents

A patent has been solicited. A Figueroa, F Gago, O Martínez, R Carballo, A Cortés, A Casas. (PCT/EP2019/081522). Compounds that selectively and effectively inhibit Hakai-mediated ubiquitination, as anticancer drugs. Fund. Prof. Novoa Santos.

## Supplementary Materials

The following are available online at https://www.mdpi.com/2072-6694/12/5/1340/s1, Table S1: List of antibodies included, Figure S1: X-ray crystal structure of the pTyr-binding domain of a Hakai dimer (PDB id. 3VK6) [35], Figure S2: Hakin-5 does not affect cell viability in epithelial tumour cell lines, Figure S3: Hakin-5 does not increase epithelial phenotype on epithelial tumour cell lines, Figure S4: Hakin-1 slightly increases apoptosis in Hakai-MDCK epithelial cells, Figure S5: Hakin-1 does not significantly affect the endogenous levels of Hakai, Figure S6: Hakin-5 does not affect the expression levels of EMT markers, Figure S7: Effect of Hakin-1 on proliferation in human cancer cells, Figure S8: Hakin-1 does not affect cell apoptosis in tumour xenograft mouse model, Figure S9: Intact cell morphology and tissue structure of liver and kidney in xenograft mice model upon treatment with Hakin-1, Figure S10: Full blots corresponding to Figure 1, Figure S11: Full blots corresponding to Figure 3, Supplementary Materials and Methods: In vitro apoptosis assay, In vivo TUNEL assay.
