# Supplementary material for: Hakin-1, a New Specific Small-Molecule Inhibitor for the E3 Ubiquitin-Ligase Hakai, Inhibits Carcinoma Growth and Progression
Source: Cancers (Basel). 2020 May 23;12(5):1340. doi: 10.3390/cancers12051340 (PMC7281109; doi:10.3390/cancers12051340)
Supplement: Supplementary file 1 [file cancers-12-01340-s001.pdf]

cancers

Supplementary Materials

Hakin-1, A New Specific Small-Molecule Inhibitor for the E3 Ubiquitin-Ligase Hakai, Inhibits Carcinoma Growth and Progression

Olaia Martinez-Iglesias, Alba Casas-Pais, Raquel Castosa, Andrea Díaz-Díaz, Daniel Roca-Lema, Ángel Concha, Álvaro Cortés, Federico Gago, and Angélica Figueroa

Supplementary Materials:

Table S1. List of antibodies included.

Antibody	Dilution	Use	Catalog nº / Provider
**Hakai**	1:1000	Western	Invitrogen 36-2800
	1:250	IHQ(P)	Invitrogen 36-2800
	1:1000	Western	BD Trans Lab 610182
**E-Cadherin**	2 ug	IP	BD Trans Lab 610182
	1:200	IF	BD Trans Lab 610182
	1:400	IHQ(P)	Cell signaling 243E10
**Cortactin**	1:1000	Western	Millipore 05-180
	1:50	IHQ(P)	Millipore 05-180
**N-Cadherin**	1:1000	Western	Abcam ab18203
	1:100	IHQ(P)	Abcam ab18203
**Vimentin**	1:1000	Western	Cell signalling D21H3
**Ki67**	1:150	IHQ(P)	Agilent M7240
**CD31**	1:100	IHQ(P)	Abcam ab 28364
**HA**	1:1000	Western	Roche 12CA5

**FLAG**	1:4000	Western	Sigma Aldrich, Clone M (F1316S)
**GAPDH**	1:5000	Western	Invitrogen 39-8600
**HRP rabbit**	1:5000	Western	GE healthcare NA934
**HRP mouse**	1:5000	Western	GE healthcare NA931
**Mouse IgG**	2 μg	IP	Santa Cruz Biotechnology sc-2025

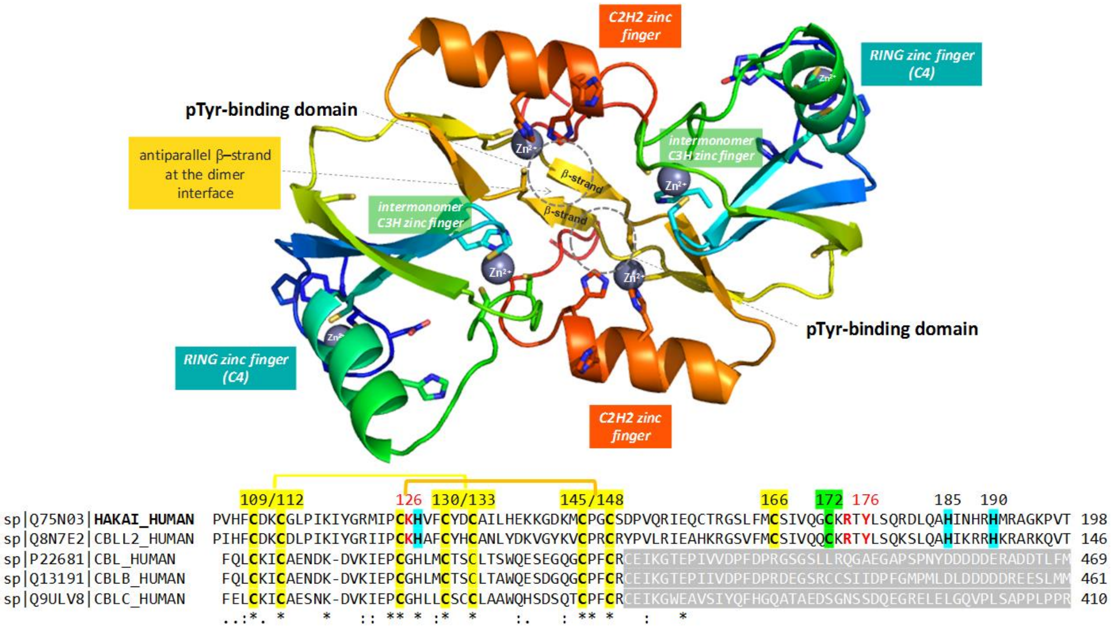

Figure S1. X-ray crystal structure of the pTyr-binding domain of a Hakai dimer (PDB id. 3VK6) [35]. Upper panel, each monomer has been rainbow-coloured to highlight the N-term (blue) to C-term directionality of the protein backbone and the symmetric nature of the active site. The side chains of Cys and His residues are displayed as sticks. The Zn^{2+} ions are represented as spheres coloured in grey. Lower panel, alignment of human Hakai (CBLL1) compared to other related E3 ubiquitin-ligases (CBLL1, CBL, CBLB and CBLC).

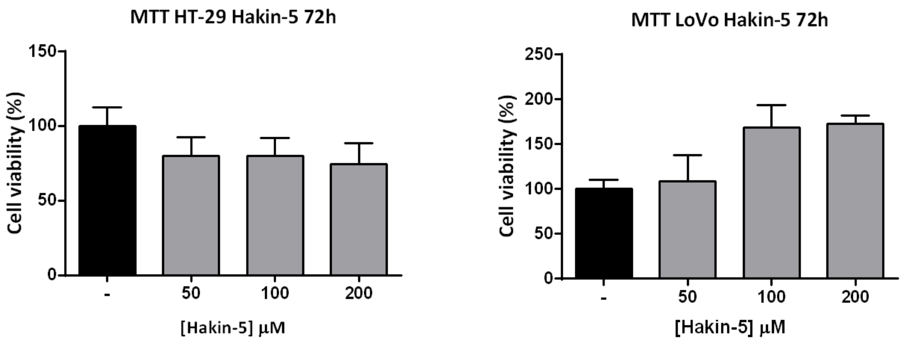

Figure S2. Hakin-5 does not affect cell viability in epithelial tumour cell lines. HT-29 and LoVo cells were treated with an increasing range of concentrations of Hakin-5 for 72 hours and cell viability was measured by means of the MTT assay. This assay was performed in 6 replicates and results are represented as mean ± SD.

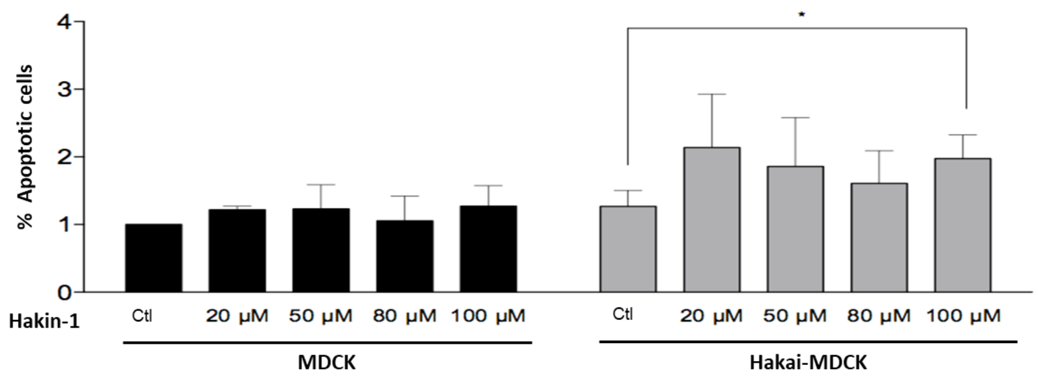

Figure S3. Hakin-1 slightly increases apoptosis in Hakai-MDCK epithelial cells. MDCK and Hakai-MDCK cells were treated with an increasing range of concentrations of Hakin-1 for 48 h, and the percentage of apoptotic cells was determined by flow cytometry using the Annexin V-FITC Apoptosis Detection kit, following manufacturer's indications. Results are represented as mean ± SD of three independent experiments.

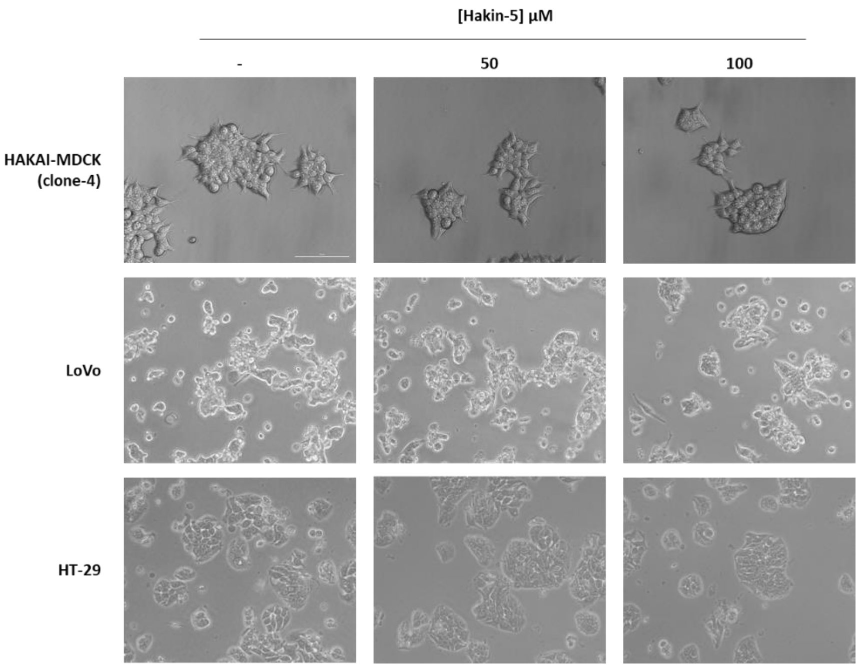

Figure S4. Hakin-5 does not increase epithelial phenotype on epithelial tumour cell lines. Phase-contrast images of Hakai-MDCK (clone-4), LoVo and HT-29 cell lines under Hakin-5 treatment. Images were obtained using a 20x objective. Scale bar, 100 µM.

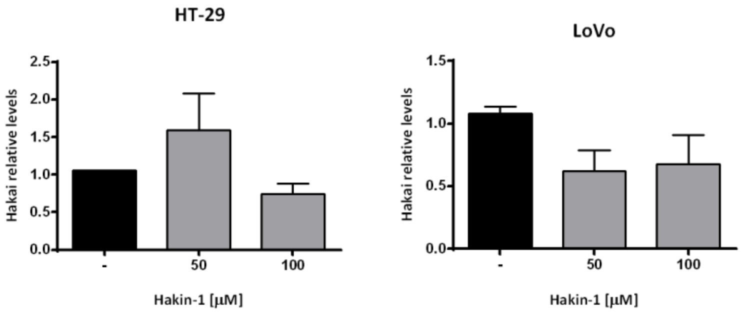

Figure S5. Hakin-1 does not significantly affect the endogenous levels of Hakai. Quantification of Hakai protein levels upon Hakin-1 treatment, analysed by Western Blot assay, in HT-29 and LoVo cells. Data are represented as mean ± SD of three independent experiments.

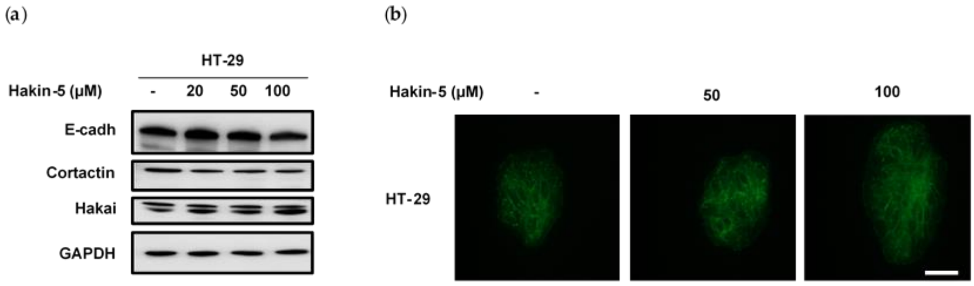

Figure S6. Hakin-5 does not affect the expression levels of EMT markers (**a**)Western blotting of E-Cadherin, Cortactin and Hakai in HT-29 cells after Hakin-5 treatment for 48 h. (**b**) Immunofluorescence of E-Cadherin in HT-29 cells treated with Hakin-5 for 48 h. Images were taken with the 40x objective. Scale bar, 250 µm.

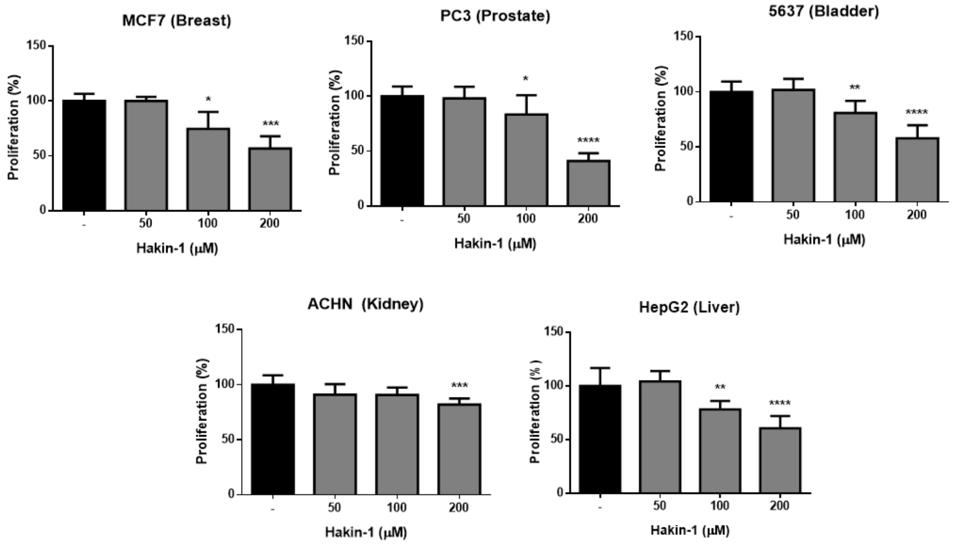

Figure S7. Effect of Hakin-1 on proliferation in human cancer cells. Breast cancer MCF7 cell, prostate cancer PC-3 cells, bladder cancer 5637 cells, renal cancer ACHN cells and liver cancer HepG2 cells were treated with Hakin-1 for 48 h and proliferation was measured by a BrdU assay as indicated in Material and Methods. Results are expressed as mean ± SD of eight replicates and experiments were repeated three times (*$p < 0.05$; **$p < 0.01$; ***$p < 0.001$).

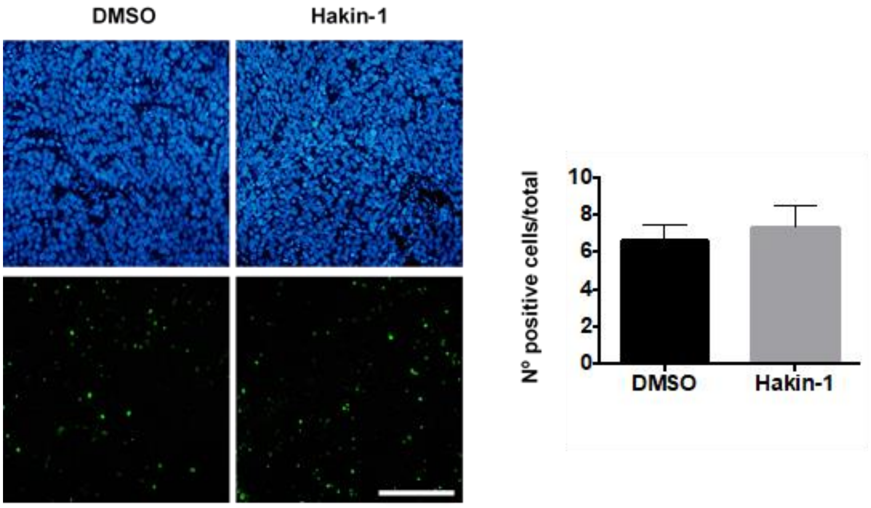

Figure S8. Hakin-1 does not affect cell apoptosis in tumour xenograft mouse model. Tunel assay was performed as indicated in Materials and Methods. A representative image is shown (left panel) and quantification of the number of positive cells is also represented (right panel) as mean ± SEM. Images were taken with 20x objective. Scale bar, 125 μm.

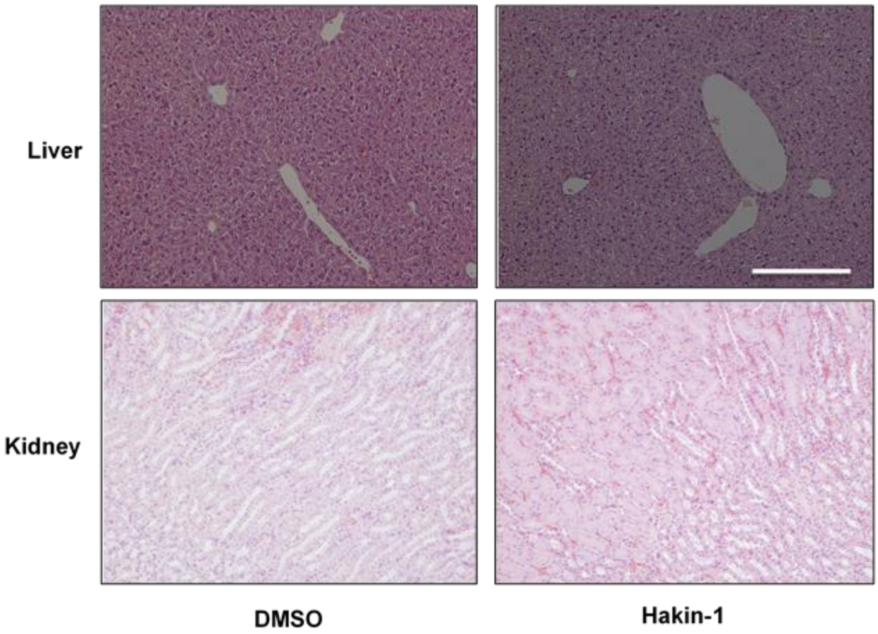

Figure S9. Intact cell morphology and tissue structure of liver and kidney in xenograft mice model upon treatment with Hakin-1. H&E staining of liver (upper panel) and kidney (bottom panel) of nude mice treated with 5mg/kg of DMSO or Hakin-1. Images were taken with a 10x objective. Scale bar, 500 μm.

Supplementary Materials and Methods

In vitro apoptosis assay

To analyse the percentage of apoptosis *in vitro*, cells were seeded in six-well culture plates at a cell density of $2x10^5$ cells per well. 24 hours after seeding, cells were treated with indicated concentrations of Hakin-1 for 48 hours. The percentage of apoptotic cells was then analysed by flow cytometry using the Annexin V-FITC Apoptosis Detection kit (Immunostep), according to manufacturer´s instructions. $10x10^3$ cells of each condition were analysed with a FACScalibur (Becton

Dickinson) flow cytometer, using the CellQuest (Becton Dickinson) software. Results are represented as mean ± SD of three independent experiments.

In vivo TUNEL assay

Tissue sections from tumours were deparaffinised and rehydrated using standard protocols. The slides were rinsed twice with PBS and treated with Target Retrieval Solution, Citrate pH 6.1 (Agilent) in microwave at 350 W for 5 min. The tissue sections were then analysed with an *in situ* Cell Death Detection Kit, Fluorescein (Roche) following the manufacturer's instructions. Then, slides were incubated with Hoechst for 5 min in darkness. The reaction was visualized under an epifluorescence Olympus microscope using 20x objective. Five representative pictures of each section were taken. The percentage of positive cells was calculated, and results are represented as mean ± SEM.

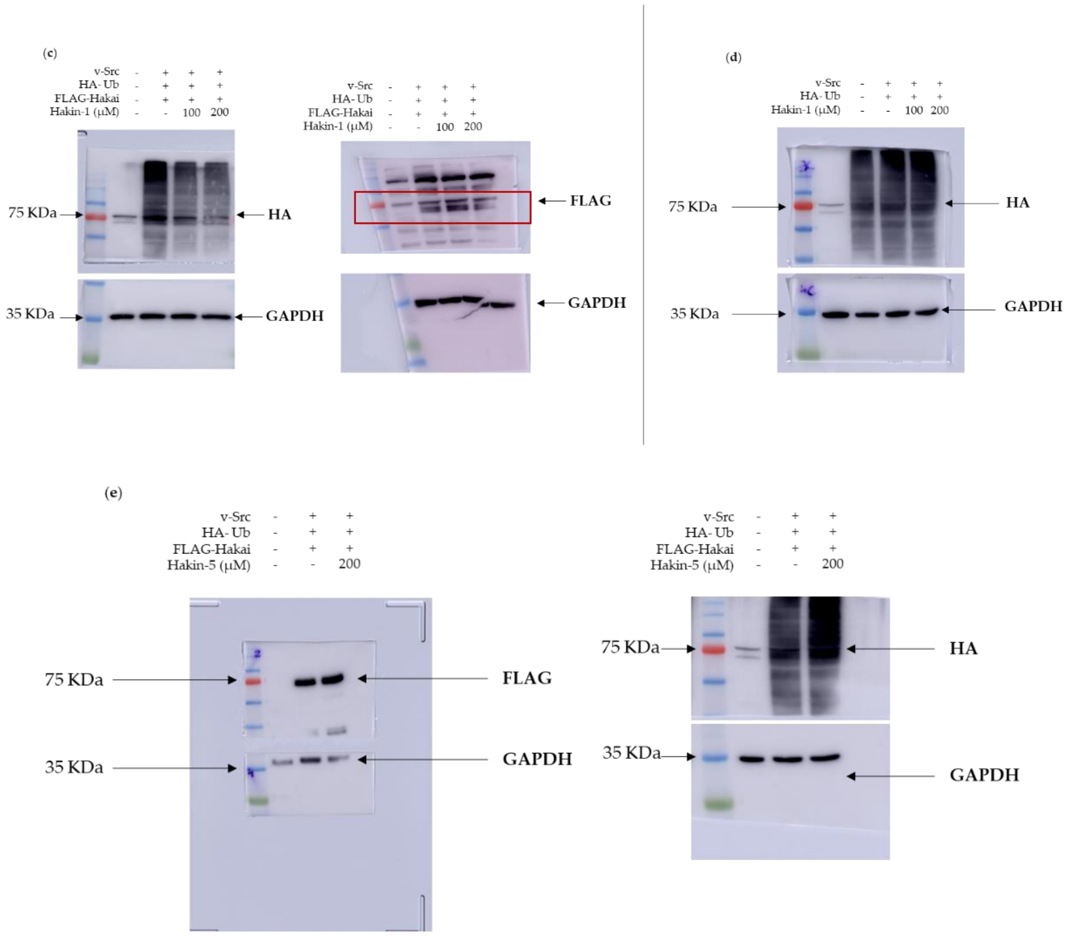

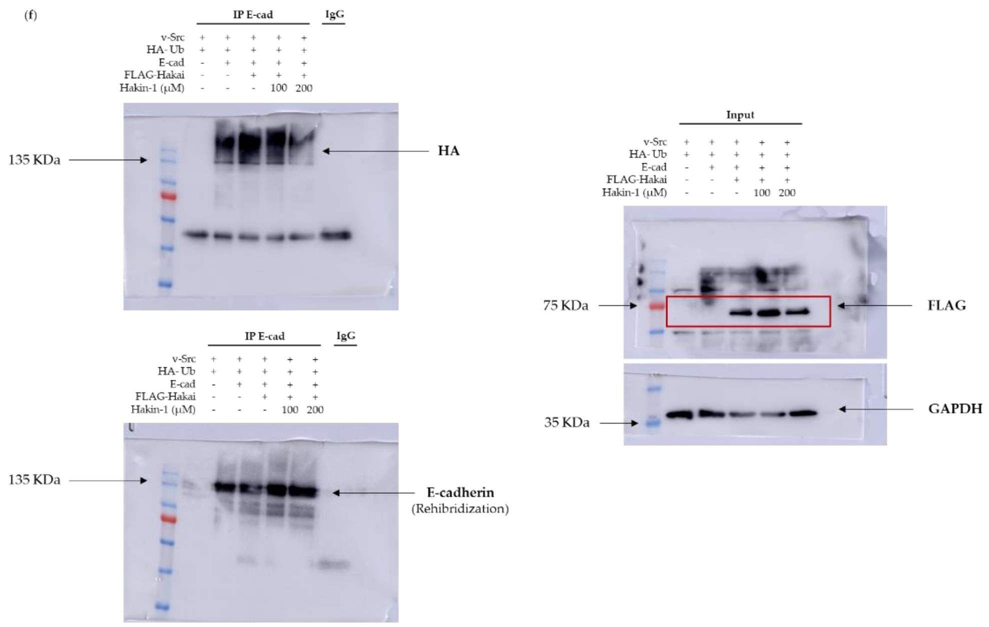

Figure S10. Full blots corresponding to Figure 1.

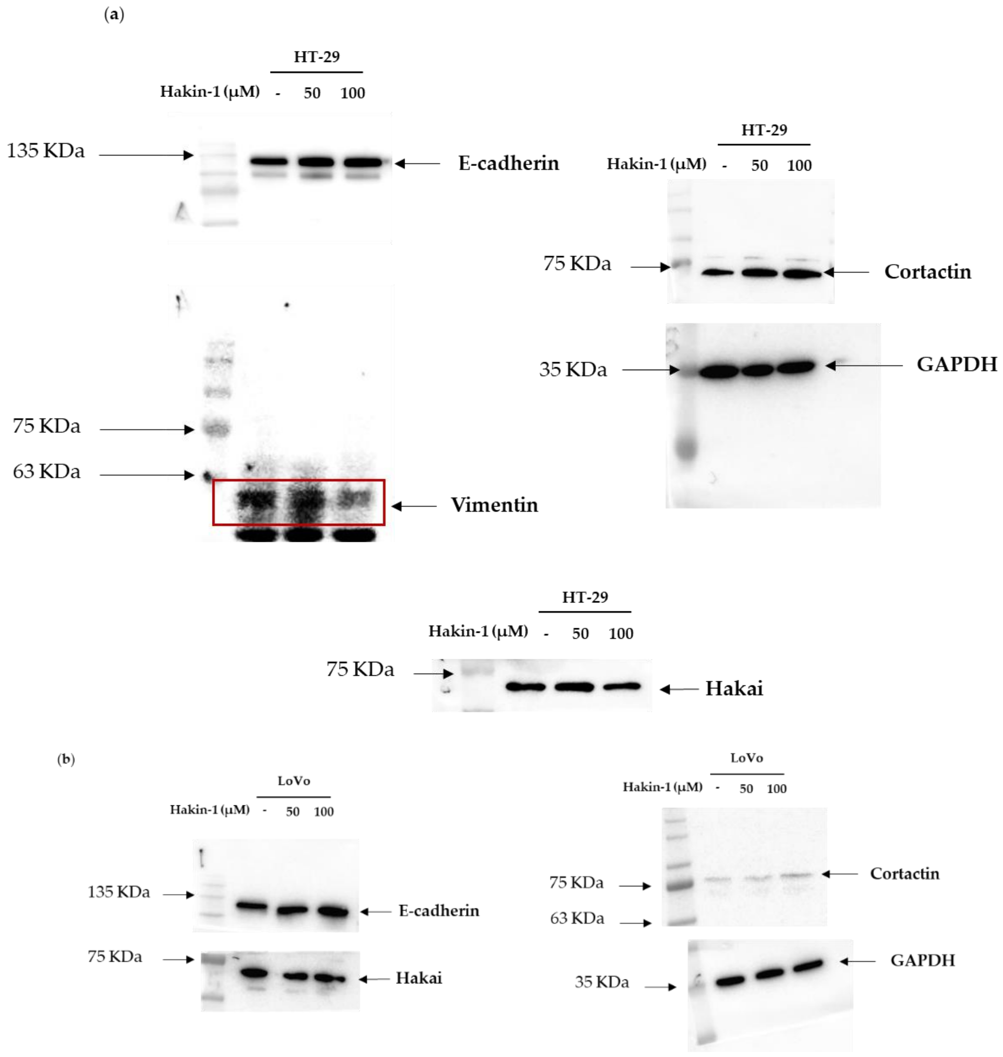

Figure S11. Full blots corresponding to Figure 3.

© 2020 by the authors. Submitted for possible open access publication under the terms and conditions of the Creative Commons Attribution (CC BY) license (http://creativecommons.org/licenses/by/4.0/).
